# Research Advances in Pheromone Biosynthesis Regulation via the PBAN Signaling Pathway in Insects

**DOI:** 10.3390/insects17050463

**Published:** 2026-04-30

**Authors:** Yu Zhang, Zhitao Liu, Yan Yi, Hong Chen, Xia Wu, Guizhi Xu, Jingjun Yang, Zhiqiang Gao

**Affiliations:** 1 College of Agricultural Science, Xichang University, Xichang 615000, China; 18819474584@163.com (Z.L.); xcc20200152@xcc.edu.cn (H.C.); zl20031003@163.com (X.W.);; 2 Panxi Crop Improvement Key Laboratory of Sichuan Province, Xichang University, Xichang 615013, China; yiyan0923@sina.cn; 3 Guangxi Forestry Research Institute, Nanning 530002, China; yangjj586@163.com

**Keywords:** insect pheromone, pheromone biosynthesis-activating neuropeptide (PBAN), pheromone biosynthesis-activating neuropeptide receptor (PBANR), secondary messenger

## Abstract

Nowadays, the application of insect sex pheromones in pest control technology has reached a relatively advanced technological maturity stage. However, the traditional research and development of sex pheromones requires a “one pest, one strategy” approach, which has drawbacks such as being time-consuming and focused on a single control target. The insect sex pheromone synthesis pathway involves multiple molecular components that work together to promote the synthesis and release of sex pheromone from the pheromone gland. Elucidating the mechanisms underlying pheromone biosynthesis offers the potential to uncover universal strategies for pheromone development, thereby improving the efficiency and effectiveness of pest management. This study arranged knowledge of the upstream regulatory pathways and summarized the structure and function of the molecular components involved. We also investigated the divergence of neuropeptides and their receptors that regulate pheromone biosynthesis among different insect species from an evolutionary perspective. Future research should integrate multi-omics, bioinformatics, structural biology, and artificial intelligence technologies to elucidate the synthesis and regulatory processes of insect semiochemicals, develop specific dsRNA and small molecule inhibitors, and accelerate the transformation and application of related molecular targets into highly effective and green pesticides.

## Simple Summary

Insects use sex pheromones to attract mates, and these pheromones have long been exploited for pest control. However, developing a pheromone method for pest control follows a ‘one pest, one strategy’ approach, making it slow and costly. Here, we explain how the pheromone biosynthesis-activating neuropeptide (PBAN) triggers pheromone production and compare this system evolutionarily across insect orders. We outline the cascade from hormone–receptor binding, via messengers such as calcium and cyclic adenosine monophosphate (cAMP), to pheromone synthesis. Our comparison suggests a notable difference: moths depend more on this pathway, whereas other insect orders appear to have evolved alternative strategies. After pheromone synthesis, we also explain how the signal is turned off. Overall, understanding these details can guide new pest control tools, such as RNA-based pesticides or small molecules that block the hormone receptor, provided that these tools target only the pest without harming beneficial insects. This knowledge supports a shift from broad-spectrum chemical insecticides towards more sustainable farming.

## 1. Introduction

Insect sex pheromones are highly sensitive and selective eco-friendly control agents that are non-toxic and pose no risk to the environment. The technology of using them for mating disruption in pest management has become well-established. However, the use of sex pheromones for pest control follows a “one pest, one strategy” principle, which leads to drawbacks such as long research and development periods and a narrow control spectrum. Consequently, its application is largely confined to a few major agricultural and forestry pests, serving as a component within integrated green pest management strategies. According to existing research, multiple molecular components are engaged in the insect sex pheromone synthesis pathway [1,2]. This paper centers on the key molecular components of the insect PBAN signaling pathway and analyzes the mechanisms by which they achieve precise regulation of sex pheromone production at the cellular, molecular, and evolutionary levels. Discovering a key conserved node in the insect sex pheromone biosynthesis pathway that governs pheromone production in multiple insects would have profound implications for the advancement of pheromone-based pest management. Specifically, it would help alleviate the current bottleneck in the discovery of original molecular targets for green pesticides—a bottleneck caused by insufficient exploration and utilization of pest-control gene resources and provide theoretical support for addressing the broader issue of insufficient innovation in green control technologies [3,4].

## 2. Pheromone Regulation Pathways in the Insect PBAN Signaling Cascade

**Figure 1:**
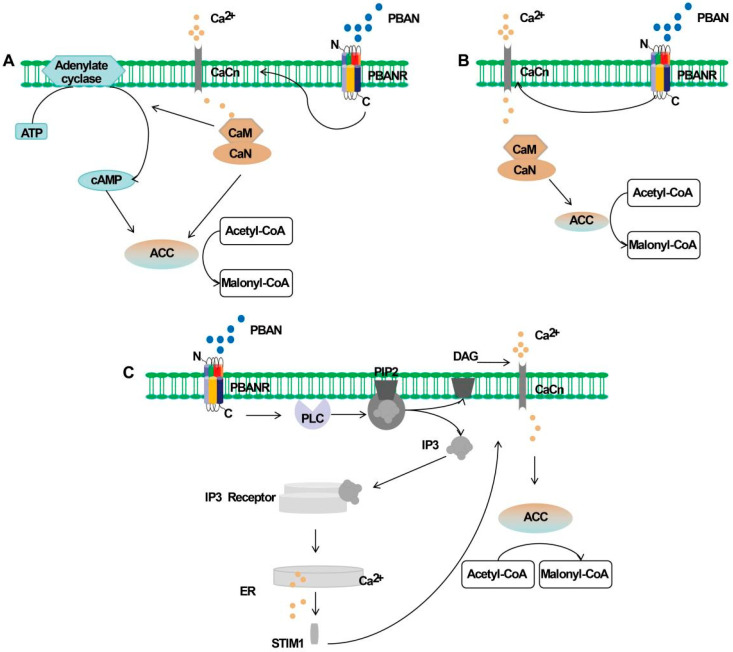
Pheromone synthesis pathways triggering different second messengers (adapted from [1,2,6]): (**A**): signaling pathway using cAMP as the second messenger; (**B**): signaling pathway using Ca^2+^ as the second messenger; (**C**): signaling pathway using IP_3_ and DAG as the second messengers (PBAN: pheromone biosynthesis-activating neuropeptide; PBANR: pheromone biosynthesis-activating neuropeptide receptor; PLC: phospholipase C; ACC: acetyl-coA carboxylase; CaCn: calcium channels; CaN: calcineurin; CaM: calmodulin; DAG: diacylglycerol; ER: endoplasmic reticulum; STIM1: stomatal interaction molecule 1; and IP3: inositol 1,4,5-trisphosphate).

The synthesis of insect sex pheromones is regulated by an upstream signaling pathway. This pathway is initiated by the pheromone biosynthesis-activating neuropeptide (PBAN), which acts as the key signaling molecule. The PBAN binds to its receptor (PBANR) located on the cell membrane of the insect pheromone gland, inducing a conformational change in the PBANR. Upon this conformational change, the PBANR triggers the production of second messengers such as cyclic adenosine monophosphate (cAMP), Ca^2+^, inositol 1,4,5-trisphosphate (IP3), and diacylglycerol (DAG) (Figure 1), which in turn activate downstream enzymes in the fatty acid synthesis pathway. This establishes a PBAN-mediated hierarchical signaling cascade that regulates insect sex pheromone synthesis, enabling the production of species-specific pheromones while also mediating other physiological functions [1,2,5].

Advances in biochemical and molecular techniques have enabled the characterization of key molecular components in the PBAN signaling pathway across 59 insect species (Table 1). These species span seven orders: Lepidoptera (35 species), including Saturniidae, Noctuidae, Pyralidae, Tortricidae, Pieridae, Plutellidae, Crambidae, Heliodinidae, Bombycidae, Gelechiidae, Nymphalidae, Sphingidae, Cossidae, and Lasiocampidae; Hymenoptera (6 species), represented by Formicidae and Pamphiliidae; Diptera (3 species), including Culicidae and Drosophilidae; Hemiptera (2 species), such as Aleyrodidae and Psyllidae; and single representatives from Coleoptera (Rutelidae), Orthoptera (Acrididae), and Thysanoptera (Thripidae) (Table 1).

## 3. Molecular Components of the Pheromone Biosynthesis-Activating Neuropeptide (PBAN) Signaling Pathway in Insects

### 3.1. PBAN, a Signaling Molecule for Insect Pheromone Production

The PBAN is a key signaling neuropeptide that regulates sex pheromone biosynthesis in many insects. It is produced in the suboesophageal ganglion and secreted into the hemolymph via the corpora cardiaca. The PBAN acts directly on pheromone glands to stimulate pheromone production or, in some cases, is transported via the ventral nerve cord to the terminal abdominal ganglion to exert its function. First identified in *Helicoverpa zea* [25], the PBAN gene in this species was subsequently cloned [52]. Although the PBAN is primarily known for regulating sex pheromone synthesis in most moths, it has also been implicated in the regulation of other pheromones in some non-lepidopteran insects, such as *Solenopsis invicta* [53].

PBAN family neuropeptides exhibit cross-species bioactivity. The PBAN receptor of *Bombyx mori* can be activated by PBANs from *Helicoverpa zea*, thereby triggering the silkworm’s sex pheromone biosynthesis pathway. Even myotropin, a signaling molecule secreted by *Locusta migratoria* (Orthoptera), can effectively stimulate sex pheromone synthesis and release in silkworms [54]. This broad regulatory effect relies on the conserved molecular basis underlying PBAN family functions. A key structural feature of PBAN/pyrokinin family peptides is the conserved C-terminal pentapeptide FXPRLamide (where X represents a variable amino acid, and “amide” indicates C-terminal amidation). This motif exhibits high structural conservation across different insect species. This unique C-terminal sequence, together with other amino acid residues of PBANs, mediates receptor binding and is essential for PBAN bioactivity. Substitution or deletion of any single amino acid within this motif significantly reduces its activity. In contrast, the N-terminal region is less conserved than the C-terminus. Amino acid changes or deletions in the N-terminal domain have relatively minor effects on PBAN activity. However, certain N-terminal residues, such as those in the hydrophobic region near the N-terminus, may contribute to neuropeptide stability or intracellular trafficking, thereby indirectly supporting its physiological function. In terms of secondary structure, the β-sheet of PBANs is often characterized by the “X-P-A-L” motif (where “X” can be amino acid residues such as serine, threonine, or valine). This structural feature contributes to the overall conformation of PBANs, conferring water solubility and enabling it to enter the insect circulatory system and reach pheromone gland receptors. These shared structural similarities not only reflect the conserved evolutionary origin of PBAN family peptides but also provide the necessary molecular basis for their functional roles across different insect species [2,55,56].

PBAN family neuropeptides exhibit structural and functional variations across different insect species, reflecting the adaptation of insects to diverse ecological and physiological demands. The PBAN/pyrokinin family is classified into two major groups: the PK/PBAN group with the conserved C-terminal FXPRLamide motif, and the PK/diapause hormone (DH) group characterized by the WFGPRLamide motif at the C-terminus [33,56]. In some insects, PBAN family peptides regulate the synthesis of pheromones other than sex pheromones. For example, the PBAN regulates trail pheromone production in *Solenopsis invicta* [53]. The PBAN stimulates aggregation pheromone (AP) synthesis, thereby influencing mating behavior in *Frankliniella occidentalis* [57]. In certain insects, PBAN family peptides regulate not only pheromone synthesis but also pheromone release. For instance, in *Trichoplusia ni*, sex pheromones are continuously produced throughout the photoperiod. The PBAN may influence pheromone precursor synthesis by regulating carbohydrate uptake, and it also stimulates the translocation of pheromones to the gland cell membrane for release during courtship, thereby playing a key role in sex pheromone transport and release [23,55].

PBAN family members retain highly conserved core domains and similar structural features. However, structural variations across different insect species enable these peptides to fulfill diverse physiological functions through distinct mechanisms and regulatory patterns. The physiological roles of the PBAN and its analogs require further characterization, and the evolutionary processes underlying such functional diversity warrant more comprehensive investigation.

### 3.2. Pheromone Biosynthesis-Activating Neuropeptide Receptor (PBANR)

Following the discovery of the PBAN, its receptor (PBANR) was identified in the pheromone glands of moths, including *Helicoverpa zea* and *Bombyx mori* [16,26,56,58]. The PBANR is a G protein-coupled receptor (GPCR) characterized by the typical seven-transmembrane domain structure [59]. In lepidopteran insects, sex pheromone biosynthesis is generally initiated upon PBAN binding to the PBANR [24,59] and the receptor activation process follows the conserved mechanism shared among GPCRs. Prior to signal transduction, the G protein α subunit associated with the PBANR is bound to GDP, which maintains the pheromone gland cells in an inactive state. PBAN binding induces a conformational change in the transmembrane domains of PBANR, exposing intracellular regions and facilitating the exchange of GDP for GTP on the G protein α subunit. This exchange activates the PBANR, transitioning the gland cells to an active state and subsequently triggering the upstream signaling pathway that regulates sex pheromone synthesis [60,61,62]. However, this canonical PBAN/PBANR pathway may not be universally required for sex pheromone regulation across all insect lineages. In a study of 24 vespid species (Hymenoptera), only PK/DH peptides and their corresponding receptors were detected, with no PK/PBAN-type peptides or receptor sequences identified, suggesting that alternative mechanisms govern sex pheromone regulation in this family [56].

### 3.3. Second Messenger Signaling Pathways

Upon conformational change in the PBANR, various types of second messengers, including cAMP, Ca^2+^, IP_3_, and DAG, are triggered (Figure 1). These messengers subsequently activate distinct enzymes in the fatty acid synthesis pathway, leading to the production of species-specific sex pheromones.

#### 3.3.1. Signaling Pathway with cAMP as the Second Messenger

In many lepidopteran insects, such as *H. zea* and *H. armigera*, cAMP functions as the second messenger to initiate de novo sex pheromone synthesis [1,2,12,13]. In this model, upon PBAN binding to the PBANR, the conformational change in the PBANR induces Ca^2+^ influx into pheromone gland cells, increasing intracellular Ca^2+^ concentration and leading to the formation of the Ca^2+^-calmodulin (CaM) complex (Ca^2+^-CaM). The formation of the Ca^2+^-CaM subsequently activates calcineurin (CaN), which in turn activates acetyl-coA carboxylase (ACC) to catalyze the conversion of acetyl-CoA to malonyl-CoA. Concurrently, the Ca^2+^-CaM regulates adenylate cyclase, promoting the conversion of ATP to cAMP. The resulting cAMP, as the second messenger, further activates ACC to facilitate malonyl-CoA production (Figure 1A). Ultimately, under the action of enzymes such as fatty acid synthase, species-specific sex pheromones are synthesized from pheromone precursor substances.

#### 3.3.2. Signaling Pathway with Ca^2+^ as the Second Messenger

In many lepidopteran insects, such as *B. mori*, *Heliothis virescens*, *Ostrinia furnacalis*, and *Spodoptera litura*, species-specific sex pheromones are also synthesized de novo using Ca^2+^ as the second messenger [2,63] (Figure 1B). Taking *B. mori* as an example, the initial steps of sex pheromone synthesis are consistent with those in insects that utilize cAMP as the second messenger. Upon PBAN binding to the PBANR, the conformational change in the PBANR induces Ca^2+^ influx into pheromone gland cells, increasing intracellular Ca^2+^ concentration and leading to the formation of the CaM. The difference lies in that, following the formation of the CaM, both CaN and calmodulin-dependent kinase II (CaMKII) are activated. Subsequently, CaMKII activates lipid storage droplet protein-1 (LSD1), which triggers lipolysis and the release of the stored pheromone precursor, Δ10,12-hexadecadienoate, from cytoplasmic lipid droplets. Concurrently, CaN facilitates the action of fatty acyl reductase (FAR), reducing Δ10,12-hexadecadienoate to 10,12-hexadecadien-1-ol, thereby completing the biosynthesis of the silkworm sex pheromone alcohol [64,65].

#### 3.3.3. Signaling Pathway with IP3 and DAG as Second Messengers

This type of signaling pathway differs substantially from the two described above. Upon PBAN-PBANR binding, phospholipase C (PLC) is activated, leading to the hydrolysis of phosphatidylinositol 4,5-bisphosphate (PIP_2_) on the membrane into inositol 1,4,5-trisphosphate (IP_3_) and diacylglycerol (DAG). In *B. mori*, IP_3_ binds to its receptor on the endoplasmic reticulum, triggering the rapid release of Ca^2+^ from intracellular stores. This process activates store-operated calcium channels on the plasma membrane, resulting in a sustained increase in intracellular Ca^2+^ concentration. The Ca^2+^ sensor stromal interaction molecule 1 (STIM1) then mediates the activation of downstream enzymes that initiate pheromone synthesis. Meanwhile, DAG can modulate calcium channels during this process, promoting Ca^2+^ influx and further elevating intracellular Ca^2+^ levels, thereby enhancing pheromone production [35,36] (Figure 1C). However, the involvement of IP_3_ and DAG as second messengers may be species-specific. For instance, in *B. mori*, DAG-mediated modulation of calcium channels does not lead to pheromone production, whereas in *Helicoverpa armigera*, DAG has been shown to stimulate pheromone production [36].

## 4. Molecular Evolution of PBAN and PBANR

**Figure 2:**
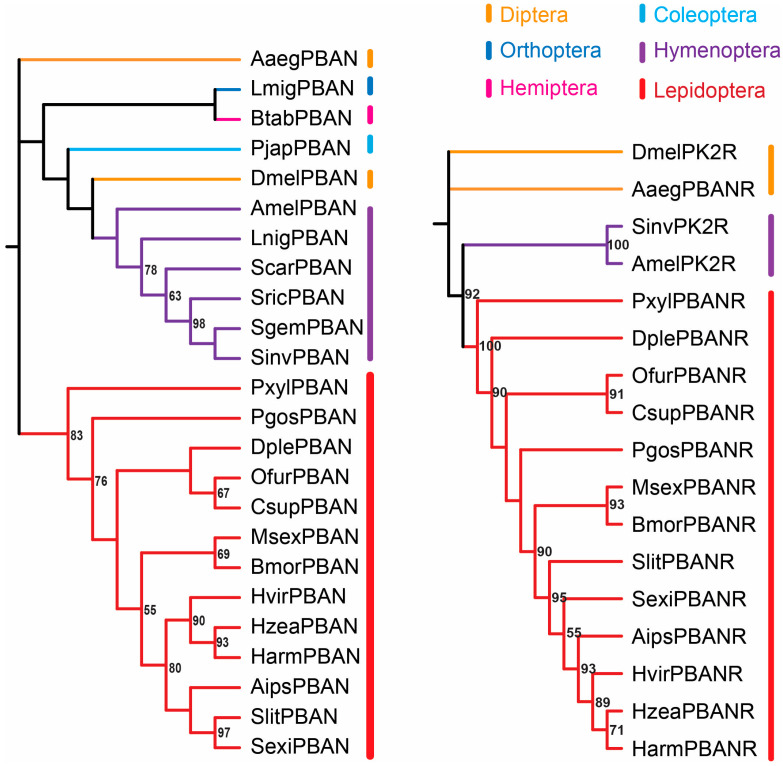
Phylogenetic tree based on PBAN and PK/PBAN receptor representative sequences. The numbers indicate bootstrap; only nodes with bootstrap ≥ 50% are marked. Amino acid sequences were aligned using MAFFT [70] with the E-INS-i strategy. The alignments are trimmed to retain conserved regions using trimAl [71]. Optimal substitution models are selected using ModelFinder [72] based on the Bayesian Information Criterion (BIC). Maximum likelihood phylogenies of PBAN were inferred using IQ-TREE [73] under the LG + G4 model for 5000 standard bootstraps. Maximum likelihood phylogenies of PK2R/PBAN are inferred using IQ-TREE under the JTT + R3 model for 5000 standard bootstraps. The tree is constructed using PhyloSuite v1.2.2. All PBAN and PK/PBAN receptor sequences are uniformly named using a four-letter abbreviation consisting of the first letter of the genus name followed by the first three letters of the specific epithet. For example, *Bombyx mori* is designated as BmorPBAN and BmorPBANR for its PBAN and receptor sequences, respectively. All phylogenetic analyses and the generation of this figure are performed by the authors using publicly available NCBI sequences. This figure is not reproduced from any previously published work.

The interaction between the PBAN and its receptor PBANR plays a crucial role in this signaling pathway. In this study, phylogenetic trees of PBAN and PK/PBAN peptide receptors were constructed using PhyloSuite v1.2.2 [66] with the “LG + G4” and “JTT + R3” models, respectively. The resulting phylogenetic analysis (Figure 2) revealed two major findings. PBAN and PK/PBAN peptides from different insect orders largely clustered into separate clades, suggesting order-specific adaptive evolution. In Lepidoptera, the PBAN and its receptor PBANR displayed a generally one-to-one pattern of divergent clustering in the phylogenetic tree, indicating that the evolutionary divergence of PBANR parallels that of PBAN and implying functional co-evolution and synergistic interaction. In a separate study, Dou and Jurenka analyzed PK/PBAN peptide receptors from 76 insect species across 15 orders and 19 families and found that only lepidopteran receptors formed a distinct monophyletic clade [67]; by contrast, receptors from other orders, such as Hymenoptera, were only partially clustered within a single clade. Collectively, the evidence from both studies supports the evolutionary conservation of the PBAN–PBANR interaction in synergistically regulating sex pheromone biosynthesis across most Lepidoptera. In non-lepidopteran insects, the absence of such coordinated regulation appears to be associated with receptor divergence within the PK/PBAN peptide family.

**Figure 3:**
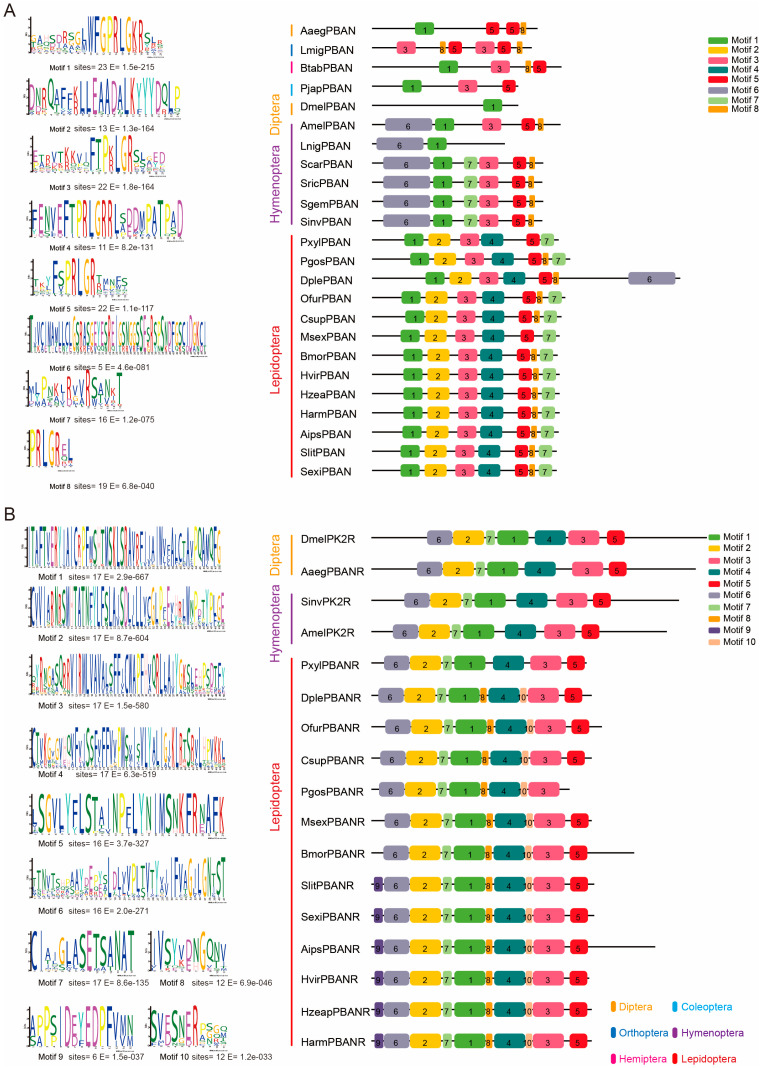
Motif structure of PBAN and PK/PBAN receptor representative sequences: (**A**): motif structure of PBAN representative sequences; (**B**): motif structure of PK/PBAN receptor representative sequences. Conserved motif analysis is performed using MEME 5.0.4 [68] with the following settings: site distribution set to “zero or one occurrence per sequence”; the number of motifs (Motif Count) is gradually increased until stable differences in conserved domain architectures are observed among different insect orders. The resulting motif patterns are visualized using TBtools v1.082 [69]. All motif analyses and the generation of this figure are performed by the authors using publicly available NCBI sequences. This figure is not reproduced from any previously published work.

We further searched for conserved motifs in representative sequences of PBAN and PK/PBAN receptors from the aforementioned insects using MEME 5.0.4 [68], which employs an expectation-maximization algorithm within a mixture model framework. The resulting motif patterns were visualized using TBtools v1.082 [69]. The analysis revealed that lepidopteran PBANs shared a conserved motif structure denoted as “1-2-3-4-5-7”. With the exception of Lasius niger, PBANs from Hymenoptera exhibited a consistent “6-1-3-5-8” motif arrangement (E-value < 0.05), whereas insects from other orders displayed more diverse motif compositions (Figure 3A). For the receptors, lepidopteran PBANRs—excluding that of *Plutella xylostella*—shared a conserved “6-2-7-1-8-4-10-3” motif structure. Notably, motifs 8 and 10 were absent in receptors from non-lepidopteran insects (E-value < 0.05). Together, these findings suggest that both PBAN and PK/PBAN receptors exhibit order-specific conservation patterns.

### Dataset Construction

We retrieved all PBAN and PK2R/PBANR sequences from the National Center for Biotechnology Information (NCBI). Where possible, we used species with both PBAN and PK2R/PBANR sequences, selecting only complete sequences to ensure alignment quality. For species with a PBAN sequence but no detectable PK2R/PBANR in NCBI (marked ‘-’ in Table 2), given that data coverage remains incomplete for some orders, we nonetheless included the PBAN sequence for broader order-level representation.

## 5. Termination of Sex Pheromone Synthesis Signals

The sex peptide (SP) is a well-recognized signal that terminates pheromone biosynthesis after mating. In certain insect species, such as *Drosophila melanogaster* and *H. armigera*, males transfer SP to females during copulation. This peptide reduces female receptivity to further mating and alters post-mating behaviors, thereby suppressing the ability to remate. Concurrently, SP inhibits pheromone synthesis in females, leading to the cessation of courtship behavior. Thus, SP functions not only to modulate post-mating female responses but also to terminate the signaling pathway by suppressing pheromone production [44,74]. In *H. armigera*, octopamine (OA) binds to its receptor OctβR and inhibits PBAN signal transduction, resulting in a reduction in intracellular calcium concentration. This inhibitory effect markedly attenuates PBAN-induced pheromone biosynthesis, thereby diminishing sex pheromone release [75]. Currently, octopamine is considered the most likely endogenous regulatory mechanism for terminating sex pheromone biosynthesis signals. Juvenile hormone (JH) in *H. armigera* not only suppresses the transcriptional level of PBANR but also enhances the responsiveness of gonadal cells to the PBAN [76]. Therefore, the overall role of juvenile hormone in pheromone production remains to be further elucidated, and its involvement in terminating pheromone biosynthesis signals may be species-specific in certain insects.

## 6. Application of Insect Pheromone Synthesis Mechanisms

In recent years, sex pheromones have achieved remarkable success in practical applications. Through strategies such as mass trapping and mating disruption, they have reduced reliance on conventional pesticides and promoted sustainable pest management [74]. In-depth research on the synthesis and regulatory mechanisms of pheromones has facilitated the large-scale production of insect sex pheromones. In industrial settings, pheromone synthesis genes are transferred into selected host organisms, and host metabolism is artificially regulated to promote the biosynthesis of specific pheromones [77,78,79]. To date, yeast species including *Saccharomyces cerevisiae* and *Yarrowia lipolytica*, as well as plants such as *Nicotiana tabacum* and *Camelina sativa*, have been developed as hosts for insect pheromone synthesis [80,81,82]. Notably, engineered oleaginous yeasts have significantly enhanced pheromone synthesis efficiency and demonstrated substantial advantages in the large-scale production of sex pheromones for various economically important crop pests, thereby providing an industrially scalable biomanufacturing platform for targeted green pest control technologies.

Researchers have successfully identified and synthesized antagonists that inhibit pheromone synthesis using approaches such as structure–activity relationship studies, molecular modeling, and biochemical assays. For example, PBAN antagonists have been shown to significantly affect the physiological processes and reproductive capacity of insects, including the rice stem borer, red imported fire ant, and *Mythimna separata* [36]. Currently, PBAN antagonists mainly consist of linear peptides and backbone-cyclic peptides (BBC), which exhibit high efficacy and metabolic stability and are capable of inhibiting sex pheromone synthesis in female moths [83].

## 7. Prospects

Insect pheromones are highly specific and environmentally friendly, yet the traditional ‘one pest, one strategy’ approach is becoming increasingly inefficient under shifting agricultural pressures [84,85]. Although recent studies have elucidated the PBAN pathway components and their conserved core structures [62,86], mechanistic and evolutionary differences across insect orders remain poorly understood [67], limiting rational design of pathway-targeted interventions.

Recent advances in omics, structural biology and AI [87] now enable systematic exploration of PBAN pathway variation. Integrative analyses and literature synthesis here suggest candidate lineage-restricted target sites within the PBANR, providing a foundation for guiding highly selective dsRNA design for RNAi-based precision control [3,4,88]. However, field application remains challenging: environmental instability, variable cellular uptake, resistance evolution [62,82], and the inherently low RNAi sensitivity of lepidopterans due to rapid dsRNA degradation [89,90] all require optimized formulations. Nanoparticle-based delivery systems, such as chitosan, lipid or carbon-based nanocarriers, can protect dsRNA from enzymatic degradation and enhance cellular uptake, representing a promising strategy to overcome these barriers [88]. As a complementary strategy, structural elucidation of PBAN-receptor interfaces combined with AI-driven screening can enable rational design of order-specific small-molecule antagonists based on motif architectures identified from the current literature. Beyond comparisons across insect orders, geographic variation within the same species represents another important but underexplored dimension. Although the available literature lacks sufficient data on whether PBAN/PBANR sequences or expression levels differ among allopatric populations that produce distinct pheromone blends, future comparative studies across geographically separated populations could help determine whether pheromone blend variation arises from evolutionary changes in the PBAN signaling pathway or from other downstream enzymes.

Collectively, based on the synthesis presented in this review, integrating multi-omics, bioinformatics, structural biology and AI will accelerate the development of specific dsRNA and small-molecule inhibitors, shifting the paradigm of green pesticide development from a species-by-species approach to modular, pathway-based precision management.

## Figures and Tables

**Table 1:** Current research progress on molecular components within the insect pheromone biosynthesis-activating neuropeptide (PBAN) signaling pathway.

Latin Nomenclature	Family	Order	The Main Molecular Components of the Signaling Pathway			References
			PBAN	PBANR	Second Messenger	
*Antheraea pernyi*	Lepidoptera	Saturniidae	+	+	-	[6]
*Antheraea mylitta*	Lepidoptera	Saturniidae	+	-	-	[7]
*Agrotis ipsilon*	Lepidoptera	Noctuidae	+	+	-	[8]
*Chlumetia transversa*	Lepidoptera	Noctuidae	+	-	-	[9]
*Helicoverpa armigera*	Lepidoptera	Noctuidae	+	+	Ca^2+^/cAMP	[10,11,12,13]
*Heliothis peltigera*	Lepidoptera	Noctuidae	+	-	-	[14]
*Heliothis virescens*	Lepidoptera	Noctuidae	+	+	Ca^2+^	[15]
*Helicoverpa assulta*	Lepidoptera	Noctuidae	+	-	-	[16]
*Mamestra brassicae*	Lepidoptera	Noctuidae	+	+	-	[17]
*Mythimna separata*	Lepidoptera	Noctuidae	-	+	-	[18]
*Spodoptera litura*	Lepidoptera	Noctuidae	+	+	Ca^2+^	[19,20]
*Spodoptera frugiperda*	Lepidoptera	Noctuidae	+	+	-	[21,22]
*Spodoptera littoralis*	Lepidoptera	Noctuidae	+	+	-	[14]
*Trichoplusia ni*	Lepidoptera	Noctuidae	+	+	-	[23]
*Helicoverpa zea*	Lepidoptera	Noctuidae	+	+	cAMP	[1,24,25]
*Spodoptera exigua*	Lepidoptera	Noctuidae	+	+	-	[26]
*Chrysodeixis eriosoma*	Lepidoptera	Noctuidae	+	-	-	[27]
*Maraca vitrata*	Lepidoptera	Pyralidae	+	+	-	[17,28]
*Ostrinia nubilalis*	Lepidoptera	Pyralidae	+	-	-	[29]
*Ostrinia furnacalis*	Lepidoptera	Pyralidae	+	+	Ca^2+^	[2] & NCBI
*Chilo suppressalis*	Lepidoptera	Pyralidae	+	+	-	NCBI
*Galleria mellonella*	Lepidoptera	Pyralidae	+	-	-	NCBI
*Cydia pomonella*	Lepidoptera	Tortricidae	+	-	-	[30]
*Pieris rapae*	Lepidoptera	Pieridae	+	-	-	[31]
*Plutella xylostella*	Lepidoptera	Plutellidae	+	+	-	[32]
*Spoladea recurvalis*	Lepidoptera	Crambidae	+	-	-	[33]
*Atrijuglans hetaohei*	Lepidoptera	Heliodinidae	+	-	-	[34]
*Bombyx mori*	Lepidoptera	Bombycidae	+	+	Ca^2+^/IP3 DAG	[35,36]
*Pectinophora gossypiella*	Lepidoptera	Gelechiidae	+	+	-	NCBI
*Danaus plexippus*	Lepidoptera	Nymphalidae	+	+	-	[37]
*Bicyclus anynana*	Lepidoptera	Nymphalidae	+	-	-	[38]
*Manduca sexta*	Lepidoptera	Sphingidae	+	+	-	NCBI
*Streltzoviella insularis*	Lepidoptera	Cossidae	+	-	-	[39]
*Holcocerus hippophaecolus*	Lepidoptera	Cossidae	+	-	-	[40]
*Dendrolimus punctatus*	Lepidoptera	Lasiocampidae	+	-	-	[41]
*Solenopsis richteri*	Hymenoptera	Formicidae	+	-	-	[42]
*Solenopsis geminata*	Hymenoptera	Formicidae	+	-	-	
*Solenopsis carolinensis*	Hymenoptera	Formicidae	+	-	-	
*Solenopsis invicta*	Hymenoptera	Formicidae	+	-	-	[42]
*Lasius niger*	Hymenoptera	Formicidae	+	-	-	NCBI
*Cephalcia chuxiongica*	Hymenoptera	Pamphiliidae	+	-	-	NCBI
*Anopheles gambiae*	Diptera	Culicidae	+	-	-	[43]
*Aedes aegypti*	Diptera	Culicidae	+	+	-	NCBI
*Drosophila melanogaster*	Diptera	Drosophilidae	+	-	-	[44,45,46]
*Bemisia tabaci*	Hemiptera	Aleyrodidae	+	-	-	[47]
*Diaphorina citri*	Hemiptera	Psyllidae	+	-	-	[48]
*Popillia japonica*	Coleoptera	Rutelidae	+	-	-	[49]
*Locusta migratoria*	Orthoptera	Acrididae	+	-	-	[50]
*Frankliniella occidentalis*	Thysanoptera	Thripidae	+	-	-	[51]

**Table 2:** Sequence used in Figure 2.

Species	PBAN ID	PK2R/PBANR ID
*Agrotis ipsilon*	CAA08774.1	AMN09327.1
*Helicoverpa armigera*	XP_063899515.1	AAW47417.1
*Helicoverpa zea*	P11159.2	AAP93921.1
*Plutella xylostella*	AEP25400.1	AAY34744.1
*Spodoptera exigua*	AXY04289.1	ABY62317.2
*Spodoptera littoralis*	AAK84160.1	ABD52277.1
*Heliothis virescens*	AAO20095.1	ABU93812.1
*Chilo suppressalis*	QPA18426.1	ALM88337.1
*Ostrinia furnacalis*	UVT35071.1	AZT88556.1
*Manduca sexta*	AAO18192.1	ACQ90219.1
*Bombyx mori*	AAB24327.1	NP_001036977.1
*Pectinophora gossypiella*	AVX48909.1	AVX48910.1
*Danaus plexippus*	XP_032528234.2	OWR48476.1
*Solenopsis invicta*	ACL35348.1	AFZ77039.1
*Solenopsis richteri*	ADI88481.1	-
*Solenopsis geminata*	ADI88478.1	-
*Solenopsis carolinensis*	ADI88480.1	-
*Lasius niger*	KMQ94925.1	-
*Apis mellifera*	NP_001104182.1	NP_001091688.1
*Drosophila melanogaster*	AAF62876.1	NP_731790.1
*Aedes aegypti*	Q16N80.1	AGT80483.1
*Popillia japonica*	KAK9758775.1	-
*Locusta migratoria*	AYC12049.1	-
*Bemisia tabaci*	UCJ19306.1	-

## Data Availability

No new data were created or analyzed in this study. All sequence data used are publicly available from NCBI. Accession numbers are listed in Table 2, and detailed sequence information is provided in Section 4.

## Abbreviations

The following abbreviations are used in this manuscript:

ACC	Acetyl-coA carboxylase
CaM	Calmodulin
CaN	Calcineurin
DAG	Diacylglycerol
ER	Endoplasmic reticulum
IP_3_	Inositol 1,4,5-trisphosphate
PBAN	Pheromone biosynthesis-activating neuropeptide
PBANR	Pheromone biosynthesis-activating neuropeptide receptor
PLC	Phospholipase C
STIM1	Stromal interaction molecule 1
